# Uvulopalatopharyngoplasty may decrease the incidence of Parkinson’s disease associated with obstructive sleep apnea

**DOI:** 10.1038/s41598-021-89205-4

**Published:** 2021-05-05

**Authors:** Heung Man Lee, Kyung-Do Han, Jeffrey D. Suh, Jae Hoon Cho

**Affiliations:** 1 Department of Otorhinolaryngology-Head and Neck Surgery, Guro Hospital, Korea University College of Medicine, Seoul, Korea; 2 Department of Statistics and Actuarial Science, Soongsil University, Seoul, Korea; 3 Department of Head and Neck Surgery, UCLA School of Medicine, Los Angeles, CA USA; 4 Department of Otorhinolaryngology-Head and Neck Surgery, Konkuk University College of Medicine, 4-12 Hwayang-dong, Gwangjin-gu, Seoul, 05030 Korea

**Keywords:** Medical research, Neurology, Risk factors

## Abstract

The purpose of this study was to investigate whether the incidence of Parkinson’s disease (PD) is increased among patients with obstructive sleep apnea (OSA) and whether surgical treatment can prevent such an increase. This was a retrospective cohort study. We analysed the claims data from the Korea National Health Insurance Service. A total of 202,726 patients who were newly diagnosed with OSA between 2007 and 2014 were included. The patients were divided into two groups: patients who underwent uvulopalatopharyngoplasty (surgery group, n = 22,742) and those who did not (conservative group, n = 179,984). The control group (n = 1,013,630) was selected by propensity score matching. They were tracked until 31^st^ December 2015. The hazard ratio of PD diagnosis (95% confidence interval) in the OSA group with respect to the control group was calculated using the Cox proportional hazard model. In the conservative group, the incidence of PD (hazard ratio 2.57 [2.32–2.85]) was significantly higher than that in the control group, while the incidence of PD in the surgery group was similar to that in the control group (hazard ratio 1.45 [0.89–2.22]). Patients with OSA are at an increased risk of developing PD, and uvulopalatopharyngoplasty may mitigate this risk.

## Introduction

Obstructive sleep apnea (OSA) is characterised by repetitive upper airway collapse during sleep, which induces frequent arousal and oxygen desaturation^1^. It is a common disease worldwide, affecting over 4% of men and 2% of women, and its prevalence increases with the prevalence of obesity^2^. OSA is associated with various symptoms such as fatigue, daytime somnolence, and headaches^1^. OSA causes serious cardiovascular complications and increases mortality^1^. Recently, various neurodegenerative disorders have also been suspected to be associated with OSA^3^.

Parkinson's disease (PD) is a typical neurodegenerative disorder that mainly affects the motor system^4^. It is characterised by tremors, rigidity, and slowness of movement at the beginning of the disease, making walking difficult^4^. As the disease progresses, dementia, depression, and anxiety occur more commonly. The characteristic pathologic findings are deposition of Lewy bodies and loss of dopaminergic neurons in the substantia nigra of the midbrain^4^. Once PD develops, it is fundamentally untreatable; attempts can only be made to improve symptoms through medication, rehabilitation, and electrical stimulation of the deep brain^4^. The prevalence of PD increases rapidly with age. The prevalence of PD in the elderly population over 60 years is about 1%, and the incidence is about 100 persons per 100,000 person-years^5^.

The relationship between PD and OSA has been steadily proposed in the literature for approximately 30 years^6^. Most studies have shown that the prevalence of OSA is high in patients with PD, probably because the upper airways of patients with PD collapse easily and their lung functions are reduced^7^. Several studies have reported that the occurrence of PD is also increased in patients with OSA^8–10^. One recent meta-analysis reported that OSA exacerbates cognitive function and motor symptoms in patients with PD, and another meta-analysis reported that OSA is an independent risk factor for PD^11,12^. Although the exact mechanism is unknown, it is presumed that the oxidative stress caused by intermittent hypoxia and sleep fragmentation in patients with OSA accelerates degenerative changes in the brain to induce the development of PD^13^. However, all these studies were based on a relatively small number of subjects, and the results differed slightly from study to study^8–10^. In addition, effects of the surgical treatment of OSA on PD incidence have not been evaluated.

Uvulopalatopharyngoplasty (UPPP) is the most commonly performed surgery for OSA^14,15^. Its success rate, based on the apnea-hypopnoea index (AHI) reduction, is not high^14^. However, UPPP can be a therapeutic option in patients who refuse or are intolerant of continuous positive airway pressure (CPAP)^14^. Therefore, it is meaningful to evaluate UPPP in terms of clinical impact, such as reduction in the incidence of PD. To demonstrate this clinical impact, long-term observational studies of large populations are required, but such studies are difficult to perform. The Korea National Health Insurance Service (KNHIS) database has recently become available for research purposes in Korea^16^. The KNHIS is a national insurer managed by the Korean government covering nearly the entire population; therefore, it can provide credible, long-term follow-up data on more than 50 million people^16^. Consequently, a retrospective cohort could be constructed by collecting data on patients with OSA as well as those who underwent UPPP.

The purpose of this study was to investigate, by using the KNHIS data, whether the incidence of PD increases among patients with OSA and whether UPPP can decrease such an increase.

## Materials and methods

This retrospective cohort study was conducted according to the STROBE guidelines^17^.

### Data source

The KNHIS is a national insurer managed by the Korean government covering nearly 100% of the Korean population and reviews both outpatient and inpatient claims, which include data on the diagnosis, procedures, prescription records, demographic information, and direct medical costs^16^. It manages the claims using a modified version of the International Classification of Diseases, 10th edition (ICD-10). Any researcher can use the KNHIS data if the study protocol is approved by the official review committee.

### Study population and design

**Figure 1:**
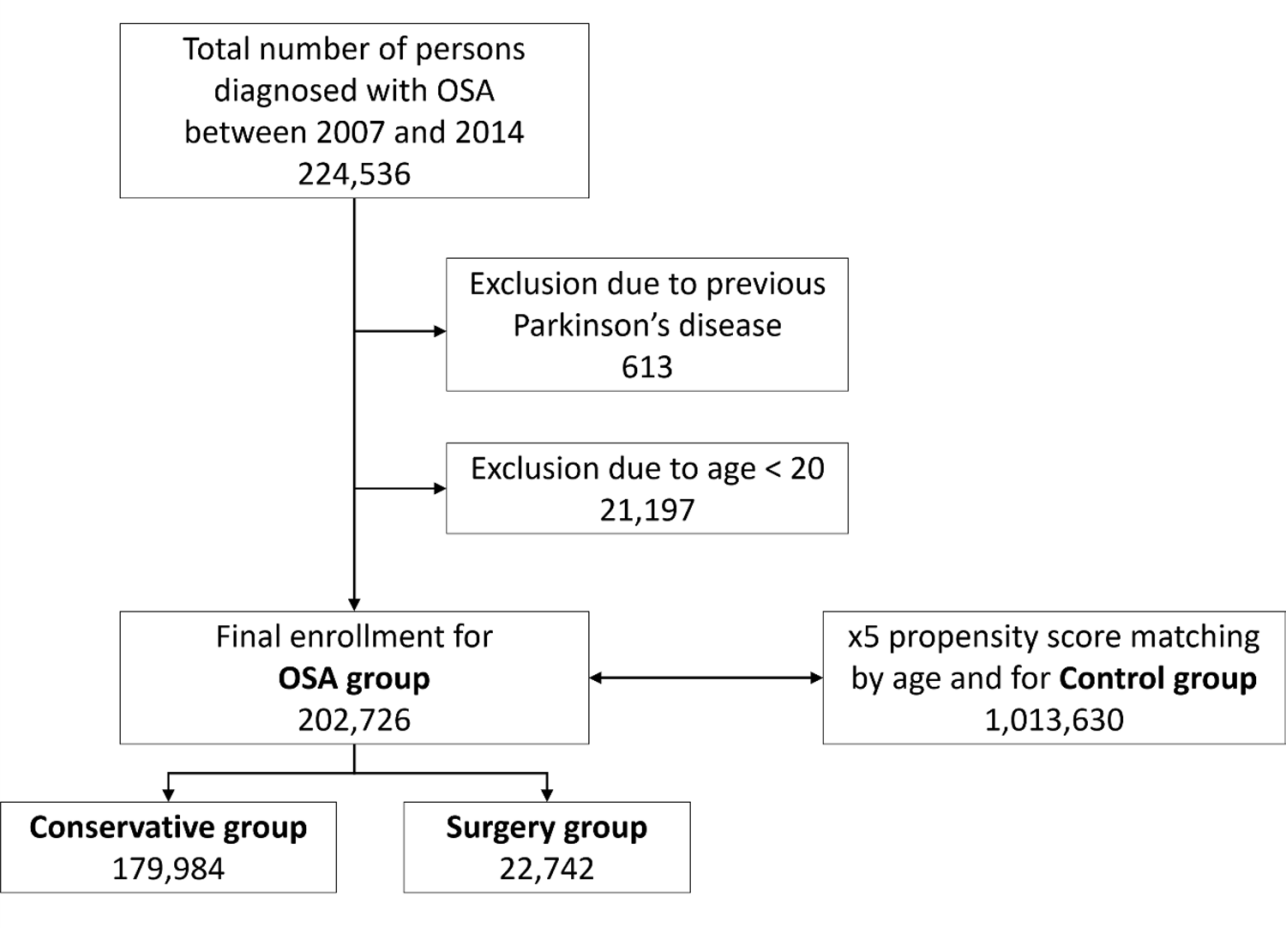
Patient enrolment flowchart.

The OSA group included subjects who were newly diagnosed with OSA (G47.30) between 2007 and 2014. Patients younger than 20 years, or those who were already diagnosed with PD, were excluded. The control group was selected from those who did not have a diagnosis of OSA, using propensity score matching by age and gender. The control group was five times larger than the OSA group. The OSA group was further divided into two subgroups: (1) the surgery group, comprising OSA patients who underwent UPPP (Q2196 or Q2197), and (2) the conservative group, comprising OSA patients who did not undergo UPPP. The patients were tracked until 31st December 2015. The primary endpoint of this study was a new diagnosis of PD, which was identified from the insurance claims data (Table 1). The flowchart shows the enrolment process for this study (Fig. 1).

**Table 1:** Working definitions derived from the insurance claims data.

Disease	Working definition
Obstructive sleep apnea	At least one claim under ICD-10 codes G47.3
Parkinson’s disease	At least one claims under ICD-10 codes G20 and registered in the National Medical Expenses Support Program
Diabetes	At least one claim per year for the prescription of anti-diabetic medication under ICD-10 codes E11, E12, E13 or E14
Hypertension	At least one claim per year for the prescription of anti-hypertension medication under ICD-10 codes I10, I11, I12, I13 or I15
Dyslipidemia	At least one claim per year for the prescription of anti-dyslipidemic medication under ICD-10 codes E78
Chronic obstructive pulmonary disease	At least one claims under ICD-10 codes J41, J42, J43 or J44
Myocardial infarction	At least one claims under ICD-10 codes I21 or I22
Stroke	At least one claim under ICD-10 codes I63 or I64
End-stage renal disease	at least one claim (R3280, O7011-O7020 or O7071-O7075) Under ICD-10 codes N18, N19, Z49, Z94, Z90 or Z992 and registered in the National Medical Expenses Support Program (V001, V003, V005 or V084)
Cancer	At least one claims under ICD-10 codes C and registered as cancer patient in the National Medical Expenses Support Program

### Data collection

We collected the following data from the KNHIS database: age (years), sex, income level (lowest quintile), and comorbidities (diabetes, hypertension, dyslipidemia, chronic obstructive pulmonary disease, myocardial infarction, and cancer). Comorbidity was defined based on the ICD code, prescription, or hospitalisation record in the claims data (Table 1).

### Statistical analysis

Comparisons between groups were made using the ANOVA or chi-square test. The cumulative incidence plot was used to identify and roughly compare the difference in PD incidence between the OSA and control groups or between the conservative and surgery groups. The incidence of PD was calculated by dividing the number of events by the person-year at risk. To determine the hazard ratio (HR) of OSA on PD, the Cox proportional hazards model was used. Three different models were applied: Model 1 was not adjusted, Model 2 was adjusted for age and sex, and Model 3 was based on Model 1, with adjustment for income level, diabetes, hypertension, and dyslipidemia^18–20^. The results are presented as HR and 95% confidence intervals (95% CIs). Statistical calculations were performed using R version 3.2.3 (The R Foundation for Statistical Computing, Vienna, Austria) and SAS version 9.4 (SAS Institute, Cary, NC, USA).

### Ethical approval

The study was exempted from approval by the Institutional Review Board of Konkuk University Hospital due to the use of publicly available data (KUH1110066). In addition, informed consent for study participants was not required because this study corresponds to secondary analysis using claim data.

## Results

Between 2007 and 2014, there were 202,726 patients newly diagnosed with OSA (OSA group). Of these, 22,742 underwent UPPP (surgery group). A total of 1,013,630 subjects were selected as the control group (Fig. 1). The mean follow-up duration was 4.8 ± 2.3 years.

### Demographic characteristics of the surgery, conservative, and control groups

In a comparison of the three groups, the surgery group had the highest male percentage (87.28%) and the lowest average age (41.8 ± 11.5 years). The lower quintile ratio of income was highest in the control group (22.43%) and lowest in the surgery group (15.44%). The prevalence of comorbidities was higher in the conservative group than in the control group. The surgery group had a lower prevalence of comorbidities than the conservative group, but the prevalence was generally higher than in the control group. The demographic data is summarised in Table 2.

**Table 2:** Demographic characteristics of patients with OSA and controls.

	Control		OSA				*p* value
			Conservative		Surgery		
	N	%	N	%	N	%	
Total number	1,013,630	100.00	179,984	100.00	22,742	100.00	
Follow-up duration (year)		4.8 ± 2.3		4.8 ± 2.3		4.4 ± 2.2	
Men	776,575	76.61	135,465	75.27	19,850	87.28	< 0.001
Mean age (year)	45.2 ± 13.3		45.6 ± 13.5		41.8 ± 11.5		< 0.001
Income lowest quintile	227,400	22.43	31,266	17.37	3512	15.44	< 0.001
Diabetes	61,262	6.04	13,683	7.60	1325	5.83	< 0.001
Hypertension	150,211	14.82	44,240	24.58	5191	22.83	< 0.001
Dyslipidemia	89,986	8.88	31,429	17.46	3102	13.64	< 0.001
COPD	99,355	9.80	28,894	16.05	3455	15.19	< 0.001
Myocardial infarction	8736	0.86	3398	1.89	229	1.01	< 0.001
Stroke	22,678	2.24	8965	4.98	510	2.24	< 0.001
Congestive heart failure	11,485	1.13	4437	2.47	315	1.39	< 0.001
End-stage renal disease	2005	0.20	683	0.38	27	0.12	< 0.001
Cancer	12,805	2.04	3026	2.41	185	1.46	< 0.001

*OSA* obstructive sleep apnea, *COPD* chronic obstructive pulmonary disease.

### Hazard ratio for Parkinson’s disease in patients with OSA

**Figure 2:**
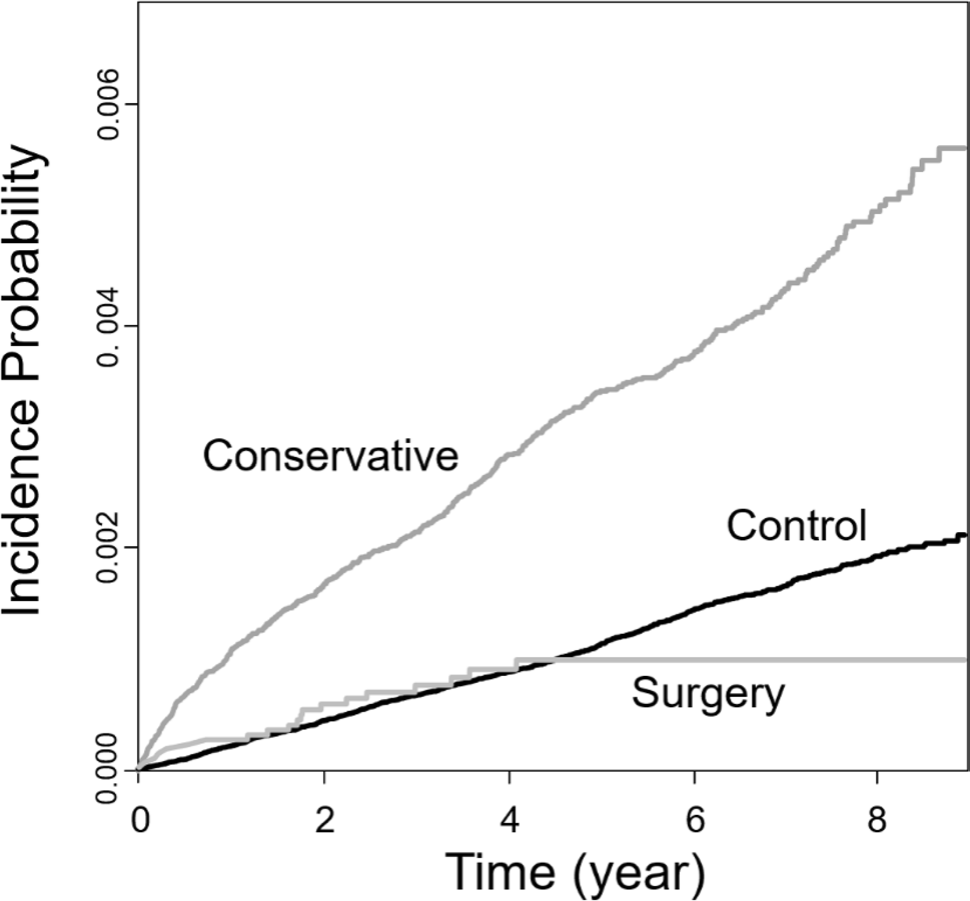
Cumulative incidence plot for Parkinson’s disease in patients with OSA. Compared to the Control group, Parkinson’s disease occurs more frequently in the Conservative group and less frequently in the Surgery group.

The cumulative incidence plot showed that PD occurred more frequently in the conservative group compared to the control group and less frequently in the surgery group (Fig. 2). The HR was higher in the conservative group than in the control group (2.84 for Model 1/2.71 for Model 2/2.57 for Model 3), and this finding was statistically significant in all three models. A comparison of the surgery group with the control group showed that the HR of a new PD diagnosis in the surgery group was lower than that in the control group in Model 1 (0.81, 95% CI 0.50–1.24), but higher than that in the control group in Model 2 (1.52, 95% CI 0.93–2.32) and Model 3 (1.45, 95% CI 0.89–2.22). However, the 95% CI included 1 for all three models, which means there was no significant difference between the surgery and control groups. These results are summarised in Table 3.

**Table 3:** Hazard ratio for Parkinson’s disease in patients with OSA.

	N	Event	Person × year	Rate	Model 1	Model 2	Model 3
Control	1,013,630	1125	4,822,203	0.23	1	1	1
**OSA**							
Conservative	179,984	572	863,159	0.66	2.84 (2.57–3.14)	2.71 (2.45–3.00)	2.57 (2.32–2.85)
Surgery	22,742	19	100,001	0.19	0.81 (0.50–1.24)	1.52 (0.93–2.32)	1.45 (0.89–2.22)

Model 1: not adjusted/Model 2: adjusted by age and sex/.

Model 3: adjusted by age, sex, income level, diabetes, hypertension, and dyslipidemia/.

*OSA* obstructive sleep apnea.

## Discussion

In this study, we found that the HR of a new PD diagnosis among patients with OSA (OSA group) was significantly higher than that in the control group. However, in patients who underwent UPPP (surgery group), the HR of PD was similar to that in the control group. Based on these results, we estimated that the incidence of PD was increased in patients with OSA and that UPPP could prevent this increase. The greatest strength of this study is that it followed up more than 1.2 million people over 4 years on average. Moreover, this is the first report on the clinical possibility of using UPPP to reduce comorbidities among patients with OSA.

Three studies have investigated the occurrence of PD in patients with OSA using the Taiwan Health Insurance data^8–10^. The initial study was conducted by Chen, and it compared 5864 patients with OSA with 23,269 controls^8^. The risk of PD in the patient group increased by 1.84 times (95% CI 1.40–2.43) after adjustment for various factors. The risk was highest in women aged 50–69 years. In a study conducted by Yeh, 16,730 patients were compared with the same number of controls, and the mean follow-up period was 5.6 years^9^. Overall, the risk of PD in the patient group increased by 1.37 times (95% CI 1.12–1.68) after adjustment. The risk was significantly increased in elderly patients over 65 years of age (30.60 times compared to those under 50 years), and there was no difference between men and women. Another study conducted by Chou compared 1944 patients with OSA with 9720 controls, and the mean observation period was 3 years^10^. The risk of PD in the patient group increased by 1.85 times (95% CI 1.02–3.35) after adjustment. When the results were further analysed by sex and age, the risk of PD was increased in men only, and it increased rapidly in elderly people over 60 years. Using the same health insurance data, all three studies showed that the incidence of PD was increased in patients with OSA, which is consistent with our findings. Therefore, we believe that our results are credible.

As mentioned in the introduction, it is presumed that intermittent hypoxia and sleep fragmentation, the major pathophysiology of OSA, induce brain degeneration and increase the occurrence of PD^13^. Although UPPP does not completely normalise the AHI in OSA patients, we believe that it might mitigate OSA and reduce the progression of PD. The incidence of PD in the surgery group was lower than that in the control group (Fig. 2). There are two main reasons for this. First, UPPP improved OSA, and second, because the average age of the surgery group was younger than that of the control group. Since the incidence of PD increases with age, it is considered that the simple incidence of PD may have decreased further in the surgery group^5^. However, when age and sex were adjusted for (Table 3), the HR for the surgery group increased to 1.52 compared to that for the control group, but the 95% CI was 0.93–2.32, which means that the risk of PD occurrence in the surgery group was not different from that of the control group.

There are no studies on the effect of CPAP or mandibular advancing devices (MAD) on the occurrence of PD in OSA patients. However, several studies have shown that when CPAP is worn in patients with PD and OSA, sleep quality, motor function, and cognition are improved, and daytime sleepiness is reduced^21–23^. In addition, MAD also improved the sleep quality of patients with PD^24^. These findings are consistent with the findings of this study that OSA treatment can partially prevent the progression of PD.

The most significant limitation of this study is the potential selection bias for the surgery group. Generally, surgery for OSA is more likely to be performed in younger, healthier, and more affluent patients due to the risks associated with anaesthesia, complications, and the cost of surgery^25^. In this study, the surgery group was younger by approximately 4 years, had a slightly higher income level, and had a lower prevalence of various comorbidities than the conservative group. Moreover, compared with the control group, the surgery group was younger and had a higher income level. Therefore, the reason for the low incidence of PD in the surgery group could be that the patients in the group were younger and healthier than those in the comparison groups, rather than the surgical effect. However, even after adjusting for various factors, the incidence of PD in the surgery group was still lower than that in the conservative group, reaching the same level as that in the control group. Thus, we can conclude this finding was not due to the measured confounding factors.

Another limitation of this study is the inability to determine the validity of the diagnosis and severity of the disease. We were unable to obtain data on AHI values ​​and symptoms from medical records. Therefore, we only defined the OSA group based on the ICD code in the claims record. In addition, since polysomnography was not performed to exclude subjects with OSA, a significant number of subjects with OSA may have been included in the control group. A further issue to be noted is that we could not determine how many patients in the conservative group were offered alternative treatments, such as CPAP or MAD. Approximately 3000 patients are prescribed CPAP annually; however, the rate of adherence to it is believed to be very low^26^. Moreover, fewer patients are prescribed MAD^16^. Considering that the number of patients in the OSA group was about 200,000, it is reasonable to assume that most patients in the conservative group did not undergo active treatment for OSA, and their condition progressed naturally.

Furthermore, the short follow-up period should be considered a limitation. The average age of the subjects ranged from 41 to 45 years. The average follow-up period was 4.8 years. Considering that PD occurs mainly in elderly individuals, the follow-up period was not sufficient. A longer follow-up period (for example, more than 10 years) would provide more definitive results. Finally, another limitation is that the analysis of various factors that can affect the occurrence of PD, such as body mass index, smoking, alcohol, occupation, and drugs were not conducted.

## Conclusion

The incidence of PD was significantly higher among patients with OSA than among controls, whereas in patients with OSA who underwent UPPP the incidence of PD was similar to that among controls. This study provides new insight indicating that UPPP may have a preventive effect on PD in patients with OSA.
